# Retrospective Analysis of Allergen Distribution Dynamics in Central Taiwan

**DOI:** 10.3389/bjbs.2023.12030

**Published:** 2023-11-14

**Authors:** Yu-Wei Tseng, Tze-Kiong Er

**Affiliations:** ^1^ Division of Laboratory Medicine, Asia University Hospital, Asia University, Taichung, Taiwan; ^2^ Department of Medical Laboratory Science and Biotechnology, Asia University, Taichung, Taiwan; ^3^ Deparment of Nursing, Asia University, Taichung, Taiwan

**Keywords:** allergy, allergen distribution, central Taiwan, prevalence, retrospective

## Abstract

**Introduction:** Allergy is a type Ⅰ hypersensitivity reaction to certain substances (allergens) such as environmental factors, food and drugs. Allergies are a significant public health issue, and therefore, understanding the distribution patterns of allergens in specific regions is important. This study aimed to retrospectively analyse allergen distribution patterns in Central Taiwan over a 5 years period (2018–2022).

**Methods:** Data of patients who had allergen sensitization testing using the OPTIGEN^®^ Allergen-Specific IgE Assay from the 1st of January 2018 to the 31st of December 2022 were reviewed retrospectively. Statistical analyses were performed to determine the prevalence and distribution of allergens in our study population.

**Results:** A total of 8,444 patients (3,784 males and 4,660 females) who attended the Asia University Hospital for allergen detection were enrolled in this study. *Dermatophagoides farina* (41.8%), *Dermatophagoides pteronyssinus* (37.9%), house dust (24.6%), cockroach mix (17.7%), crab (12.6%), clam (9.8%), shrimp (9.1%), cat dander (8.1%), pig weed (8%) and peanut (7.8%) were identified as the ten allergens that most commonly induced sensitization in our study population. Additionally, crab, clam, shrimp, peanut and beef were the five most common food allergens.

**Conclusion:** In summary, our findings contribute significantly to the knowledge on allergen distribution in Central Taiwan. Our identification of prevalent allergens may contribute to an improved understanding of the epidemiology of allergies in this region.

## Introduction

Allergic reactions are common and are characterized as Type I hypersensitivity responses to specific substances known as allergens. These allergens encompass a range of triggers, including environmental factors, food items, drugs and so on. Allergic reactions can manifest in various forms, such as asthma, rhinitis, eczema and food allergies, significantly impacting the quality of life for affected individuals. Moreover, the frequency of asthma and other atopic diseases, such as allergic rhinitis, atopic dermatitis and food allergies has significantly increased in recent decades [1]. Allergic diseases present a substantial global burden, contributing significantly to morbidity worldwide and placing a considerable strain on the health and medical systems of both developed and emerging economies [2]. Furthermore, there is a notable rise in the complexity and severity of allergic diseases, particularly asthma, among children, and young adults [3].

Allergens rank among the most significant environmental exposures. Notable indoor allergens associated with asthma comprise dust mites, cockroaches, animal dander, and mold [4, 5]. Furthermore, respiratory allergic diseases can be triggered by other reported environmental factors, which encompass cats, dogs, exposure to secondary tobacco smoke, and air pollution [6]. The identification of allergens that affect these patients can aid in avoiding allergic responses, assisting in treatment decision-making, and enhancing the prediction of prognosis [7].

The evaluation of sensitization to various allergens can be conducted through *in vivo* or *in vitro* assessments. In comparison to the skin prick test (*in vivo*), *in vitro* assays offer several advantages, including high specificity, the absence of medication cessation requirements, enhanced safety, non-invasiveness, the potential for long-term sample maintenance, and the ability to perform the test without limitations such as age or dermographism [8]. Continuous development of multiple allergen simultaneous tests (MAST) has been observed among commercially available *in vitro* allergy tests. This development has been characterized by advancements in reducing serum consumption, achieving shorter turnaround times, and incorporating a broader spectrum of allergens within the test [9].

Understanding the distribution patterns of allergens in specific regions is crucial for developing effective preventive measures, optimising allergy management strategies and improving public health outcomes. The objective of this study was to conduct a retrospective analysis of allergen distribution patterns in Central Taiwan over a 5 years period from 2018 to 2022. By assessing a wide range of allergens, we aimed to provide valuable insights into the prevalence of allergens in the region. The findings from this study will contribute to the understanding of the epidemiology of allergies in Central Taiwan and provide a foundation for the development of evidence-based public health interventions and targeted allergy management strategies.

## Materials and Methods

### Study Design and Study Population

A retrospective study was conducted on a cohort of patients who attended to the Asia University Hospital for allergen detection between 1 January 2018 and 31 December 2022. The study population consisted of 8,444 individuals, including 3,784 males and 4,660 females. We sourced our data from the patient database where the OPTIGEN^®^ Allergen-Specific IgE Assay (MAST, OPTIGEN) was employed for testing. The prevalence of different allergens was determined by calculating the percentage of patients positive for each allergen category.

### OPTIGEN^®^ Allergen-Specific IgE Assay

The OPTIGEN^®^ assay was utilized for the semiquantitative determination of allergen-specific IgE concentrations. The principle of operation for the OPTIGEN Allergen-Specific IgE Assay is based on Chemiluminescence Immunoassay (CLIA). MAST pette chambers encompass 36 different allergens, including latex, avocado, pork, beef, milk, cheddar cheese, shrimp, crab, clam, codfish, tuna, peanut, soybean, wheat (food), brewer’s yeast, egg yolk, egg white, chicken feathers, Bermuda grass, black willow, eucalyptus, Japanese cedar, white mulberry, pigweed, ragweed mix I, Timothy grass, Alternaria, Aspergillus, Cladosporium, Penicillium, cat dander, dog dander, housedust, cockroach mix, mite DF, and mite DP [7]. MAST pette chambers encompassed 36 different allergens. The obtained results were interpreted on a scale ranging from classes 0 to 4. A class ≥1 was interpreted as positive.

### Statistical Analysis

The rate of different allergens was determined by calculating the percentage of patients that were positive for each allergen category. Statistical analyses were performed to determine the prevalence and distribution of allergens in our study population. The results were analysed using the software R version 4.0 [10]. A chi-square test was used to determine the *p*-values. For the trend of prevalence, the Cochran-Armitage trend test was applied. Statistically significant values were considered as *p*-values of <0.05.

## Results

### Prevalence Changes of Allergens

**FIGURE 1:**
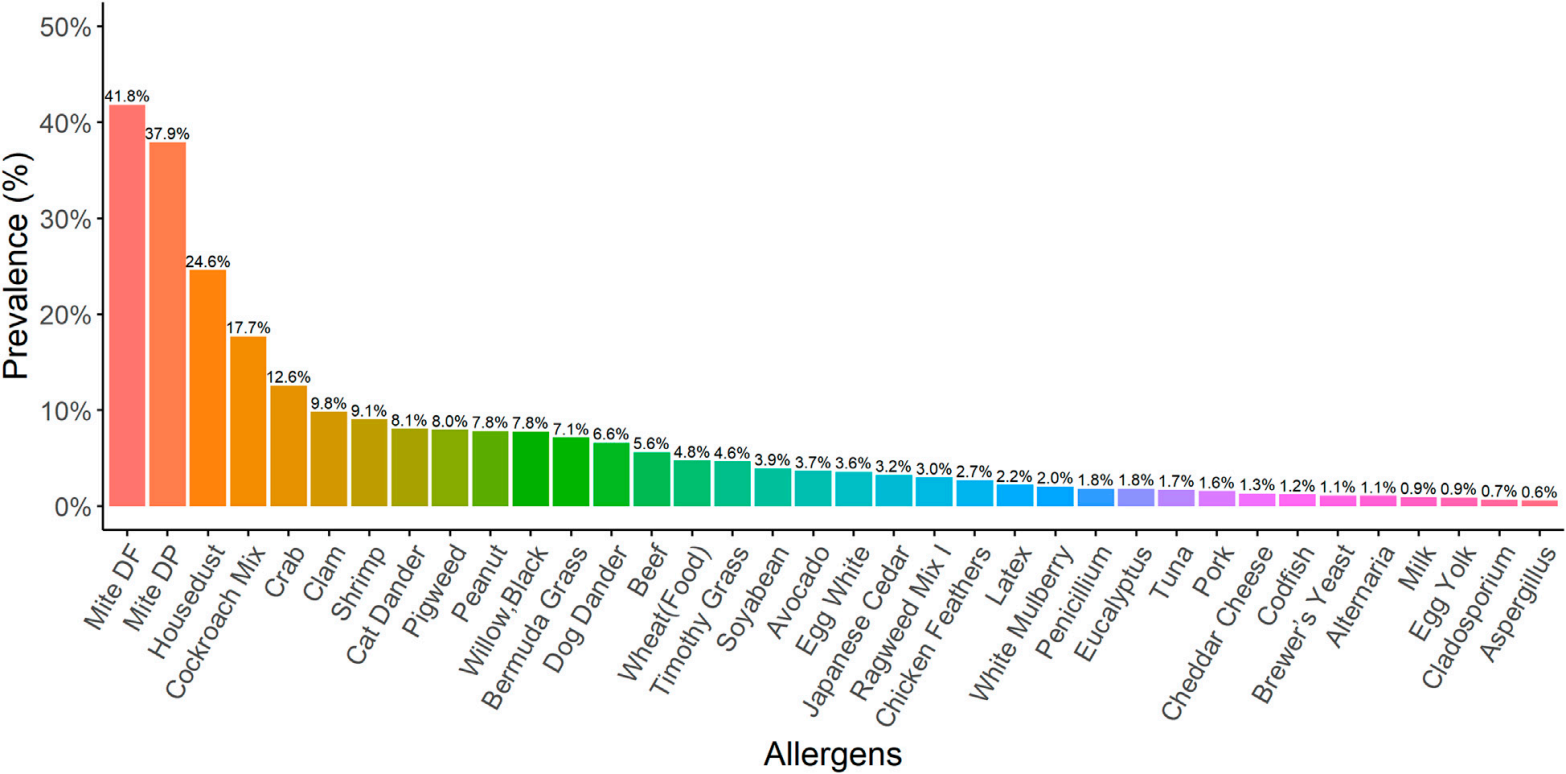
The prevalence for different kinds of allergens In our study population, the ten allergens most commonly identified as inducing sensitisation were *Dermatophagoides farinae* (41.8%), *Dermatophagoides pteronyssinus* (37.9%), house dust (24.6%), cockroach mix (17.7%), crab (12.6%), clam (9.8%), shrimp (9.1%), cat dander (8.1%), pigweed (8%), and peanut (7.8%).

A total of 8,444 patients (3,784 male and 4,660 female) were analysed in this study. Patients received the OPTIGEN^®^ Allergen-Specific IgE Assay. *Dermatophagoides farinae* (41.8%), *D. pteronyssinus* (37.9%), house dust (24.6%), cockroach mix (17.7%), crab (12.6%), clam (9.8%), shrimp (9.1%), cat dander (8.1%), pigweed (8%) and peanut (7.8%) were the ten most common allergens identified that induced sensitisation in our study population (Figure 1). Dust mites were also identified as significant allergens, particularly in indoor environments. Additionally, crab, clam, shrimp, peanut and beef were the five most common food allergens.

**FIGURE 2:**
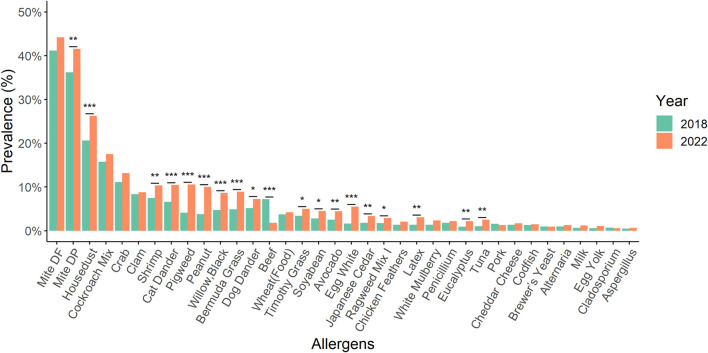
By using a chi-square test to compare the prevalence of various allergens with that of 2018 and 2022, we observed statistically significant increases in the prevalence of *D. pteronyssinus*, house dust, shrimp, cat dander, pigweed, peanut, black willow, Bermuda grass, dog dander, Timothy grass, soybean, avocado, egg white, Japanese cedar, Ragweed mix I, latex, Eucalyptus, and tuna. Conversely, the prevalence of beef allergen showed a statistically significant decrease. *: *p*-value < 0.05; **: *p*-value < 0.01; ***: *p*-value < 0.001.

**FIGURE 3:**
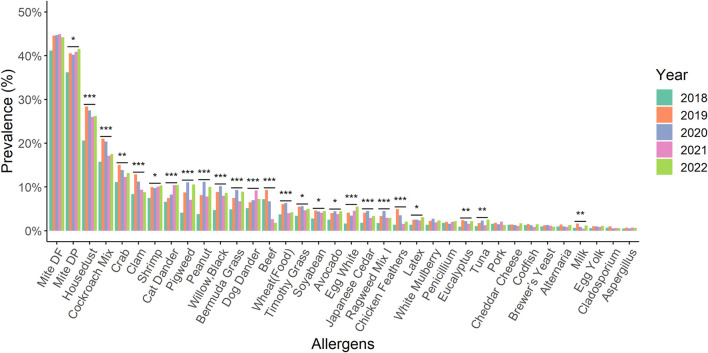
A comparison of the prevalence for different kinds of allergens from 2018 to 2022 Within 2018 and 2022, there were statistically significant differences in the prevalence of 25 types of allergens compared by the chi-square test. *: *p*-value < 0.05; **: *p*-value < 0.01; ***: *p*-value < 0.001.

The prevalence of allergens such as *D. pteronyssinus*, house dust, shrimp, cat dander, pigweed, peanut, black willow, Bermuda grass, dog dander, Timothy grass, soybean, avocado, egg white, Japanese cedar, Ragweed mix I, latex, Eucalyptus, and tuna showed statistically significant increases between 2018 and 2022 (Figure 2). Intriguingly, beef allergen statistically significant decreased compared to 2018 and 2022. Furthermore, the prevalence of 25 types of allergens showed statistically significant differences between 1 January 2018 and 31 December 2022 (Figure 3).

### Prevalence Changes of Allergens Between Male and Female

**FIGURE 4:**
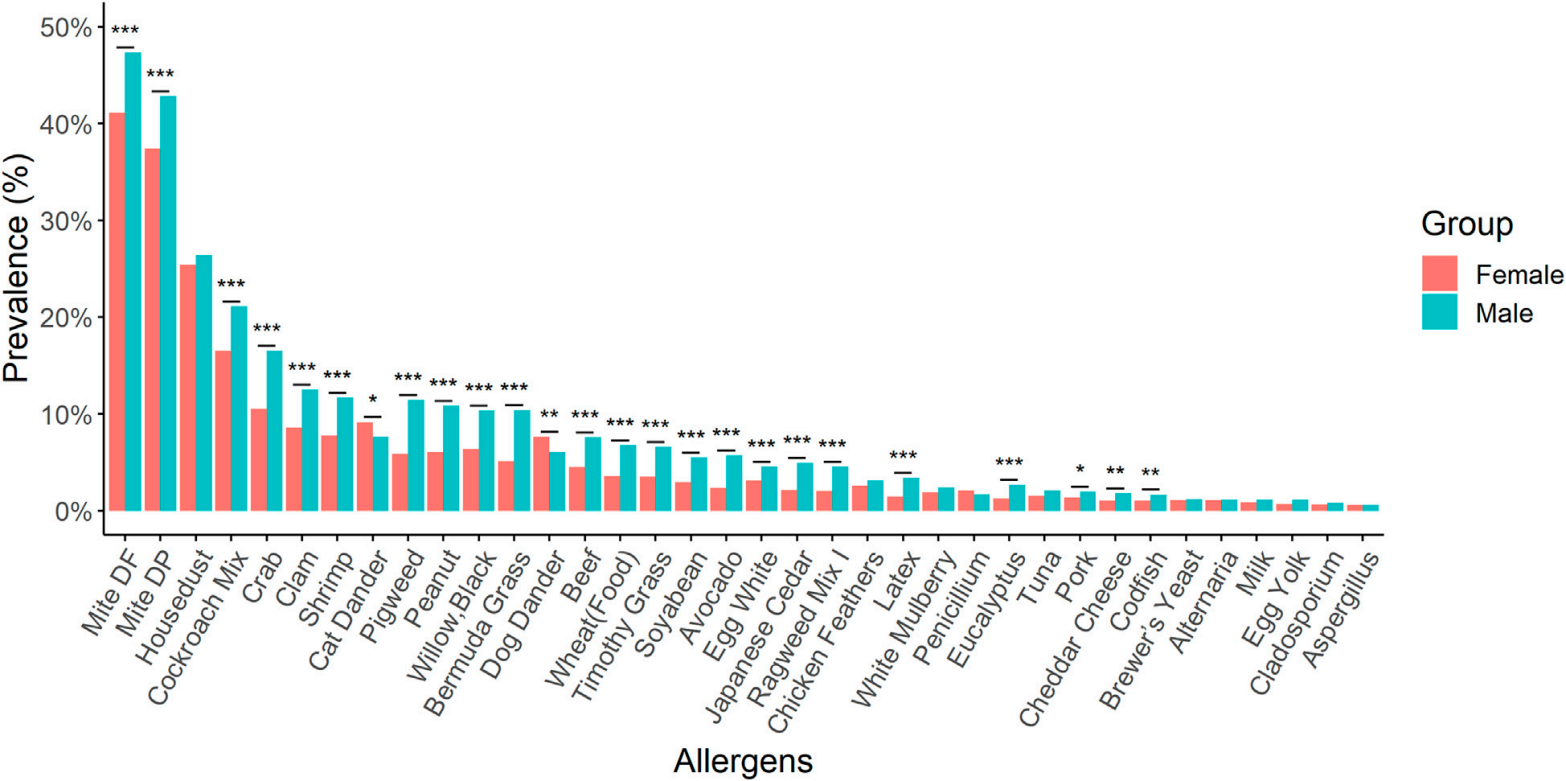
A comparison of the prevalence for different kinds of allergens between males and females. Males exhibited a statistically higher prevalence for 23 different allergens. Conversely, females demonstrated a significantly greater sensitisation rate to allergens specific to dog and cat dander compared to males. *: *p*-value < 0.05; **: *p*-value < 0.01; ***: *p*-value < 0.001.

Notable variations in the prevalence changes of allergens were observed between males and females in our study population. For instance, 23 types of allergens such as *D. farina*, *D. pteronyssinus*, cockroach mix, crab, and others showed statistically higher prevalence among males. On the other hand, females had a statistically higher dog dander and cat dander allergen-specific sensitisation rate than males (Figure 4).

### Prevalence Changes of Allergens Between Different Ages

**FIGURE 5:**
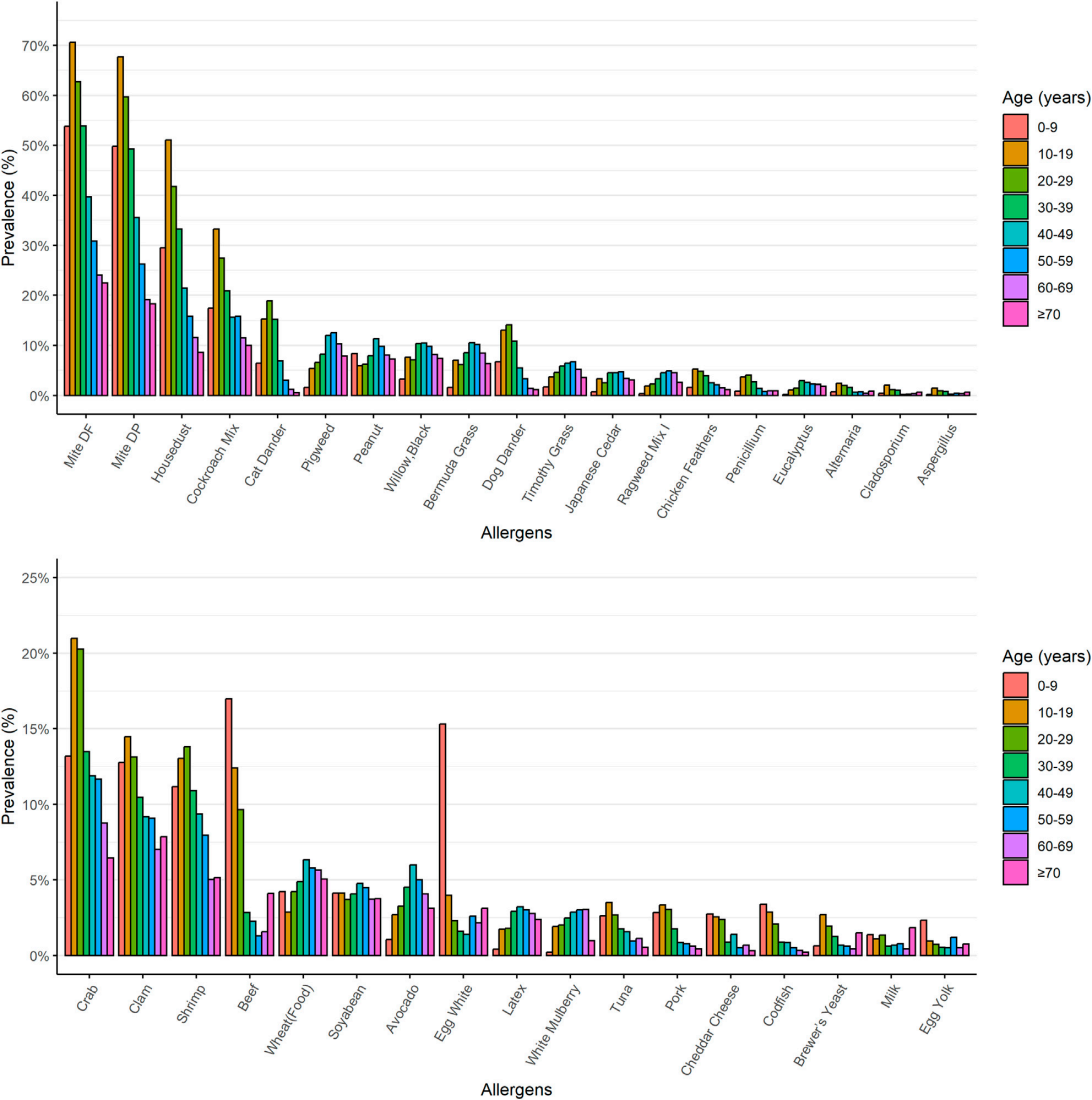
The prevalence of the most common allergens in eight different age groups of patients. The prevalence of the most common allergens was charted across eight distinct age categories of patients. The upper portion of the chart displays non-food allergen categories, while the lower portion represents food allergen categories.

We revealed the distribution patterns of each allergen across various age groups. Figure 5 illustrates the percentages of the most prevalent allergens among eight distinct patient age categories. In children aged 0–9 years, the five predominant allergens that induced sensitization were: *D. farina* (53.8%), *D. pteronyssinus* (49.8%), house dust (29.5%), cockroach mix (17.4%), and beef (17.0%). For patients aged 10–19, the top allergens were: *D. farina* (70.6%), *D. pteronyssinus* (67.7%), house dust (51.0%), cockroach mix (33.2%), and crab (21.0%). For patients aged 20–29, the leading allergens were: *D. farina* (62.7%), *D. pteronyssinus* (59.7%), house dust (41.8%), cockroach mix (27.5%), and crab (20.3%). In the 30–39 age group, cat dander (15.2%) emerged as one of the top five allergens, joined by *D. farina* (53.9%), *D. pteronyssinus* (49.3%), house dust (33.2%), and cockroach mix (20.9%). Patients aged over 40 primarily showed allergic reactions to *D. farina*, *D. pteronyssinus*, house dust, cockroach mix, and pigweed. Notably, the elderly aged 70 and above showed allergic reactions to *D. farina*, *D. pteronyssinus*, cockroach mix, house dust, and clam.

### Trend in the Prevalence of Allergens Over a Five-Year Period

**FIGURE 6:**
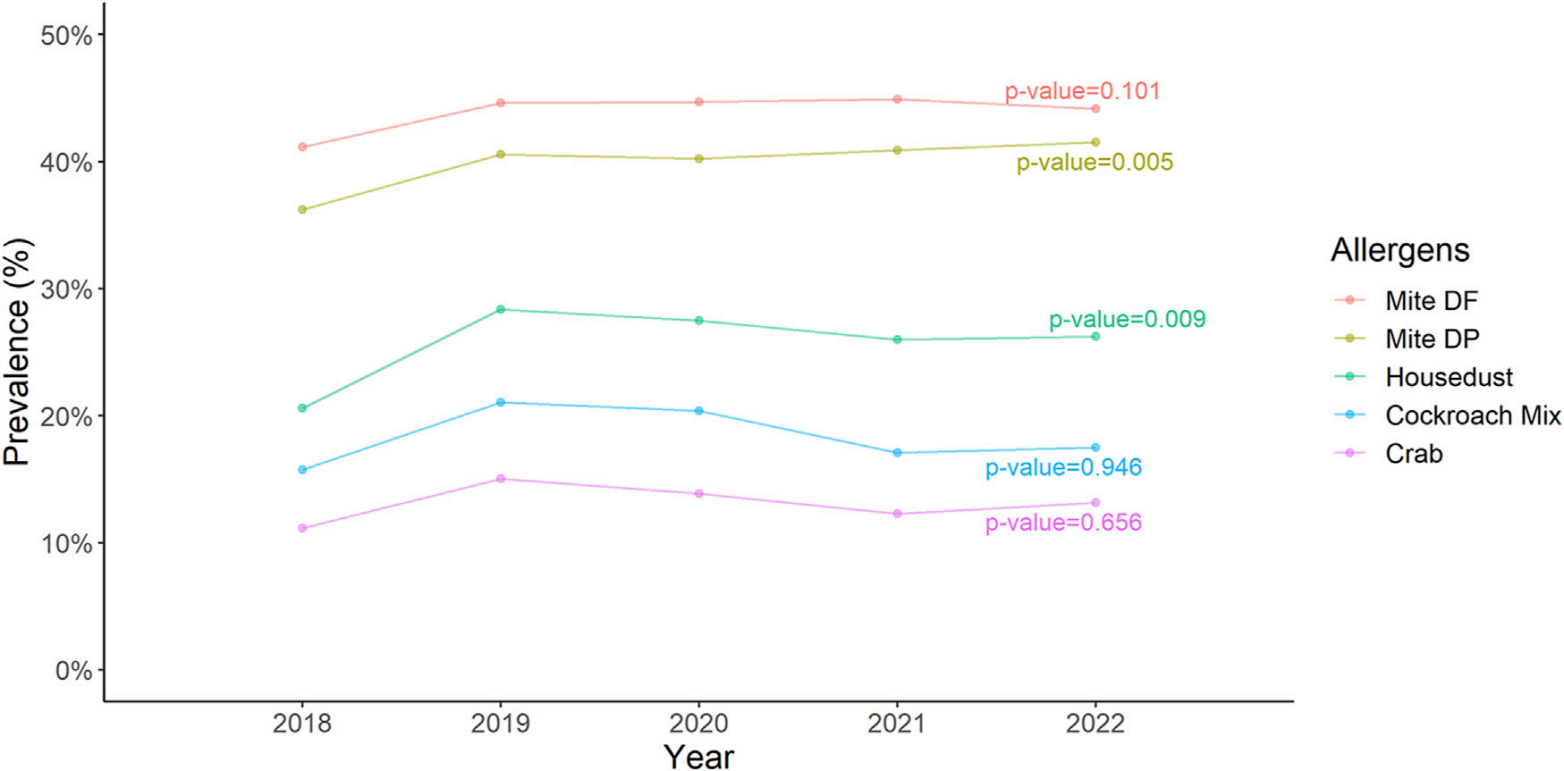
The trend of the prevalence of five most common allergens over a 5 years period Cochran-Armitage trend test indicates a significant change in trend of *Dermatophagoides pteronyssinus* and housedust.

In the analysis spanning 5 years, we observed a notable trend in the prevalence of two allergens: *D. pteronyssinus* and house dust, with the changes being statistically significant at a *p*-value less than 0.05 (Figure 6).

## Discussion

The findings of this retrospective study provide valuable insights into the distribution patterns of allergens in Central Taiwan over a 5 years period (2018–2022). Our results revealed that *D. farina*, *D. pteronyssinus*, house dust and specific food allergens were the most commonly reported allergens in Central Taiwan.

House dust mites (HDMs) play a significant role in causing allergic diseases [11]. The main types of house dust mites include *D. pteronyssinus*, *D. farinae*, *Euroglyphus maynei*, and *Blomia tropicalis* [12]. *D. pteronyssinus* and *D. farinae* are the primary sources of allergens among house dust mite species, significantly affecting allergic patients worldwide. On the other hand, *B. tropicalis* thrives in very humid and hot climates, making it limited to tropical and subtropical regions [13]. Allergic diseases are predominantly caused by HDM allergens, with approximately half of individuals with allergies experiencing an allergic reaction to these specific allergens [14, 15]. There is growing evidence showed that HDM allergy significantly contributes to asthma globally, and long-term avoidance have shown effectiveness in preventing sensitization and reducing the occurrence and intensity of respiratory diseases [16]. HDMs have consistently been identified as one of the most prominent factors associated with asthma in individuals of all age groups, including children, adolescents, and adults [17].

Melen et al demonstrated that males exhibited a higher prevalence of sensitization to airborne allergens compared to females across all age groups [18]. We indeed demonstrated that *D. pteronyssinus* and *D. farina* were statistically higher in males. Previous studies has demonstrated a correlation between the levels of HDM found in homes and the manifestation of asthma symptoms in both children and adults sensitized to HDM [19, 20]. Therefore, it should be regarded as a significant concern and a probable source contributing to the exacerbation of asthma symptoms. Additionally, a study implicated that the child’s mattress exhibited the highest percentage of HDM among the household regions [21]. Therefore, clinicians should adopt a practical approach to mitigating mite allergen exposure and recommend sensitized patients to implement a comprehensive set of measures aimed at achieving the greatest possible reduction in exposure [17]. In a multi-center study conducted by Guan et al., it was shown that increased frequencies of sensitization to HDM allergens were associated with a heightened risk of developing allergic diseases [22]. In a study conducted by Sun et al. in Taiwan, it was revealed that the concentration of HDM exhibited a seasonal variation, with the highest concentrations observed from July to November and gradually decreasing from December to June in central Taiwan [21]. The authors indicated that *D. pteronyssinus* was found to be the dominant species, accounting for 77% of the identified mites, followed by *D. farinae*, which comprised 13% of the species identified. A large-scale population-based study conducted by Huang et al demonstrated that mite sensitization had the highest prevalence among all tested allergens in Taiwan [4]. The authors revealed that HDMs played a crucial role as triggers for not only asthma and allergic rhinitis but also atopic dermatitis. Indeed, *D. farinae* and *D. pteronyssinus* were found to be the most common allergens in our study population. Figure 2 showed that *D. pteronyssinus* and house dust had increased from 2018 to 2022. Furthermore, the change in the trend of the prevalence of *D. farinae*, *D. pteronyssinus* and house dust over a 5 years period is statistically significant (Figure 6).

Food allergy has emerged as a significant public health concern over the past decade, threatening the lives of affected individuals. It is estimated that the prevalence of food allergy is approximately 3%–5% in adults and can reach up to 8% in children [23]. This increase in prevalence has prompted increased attention and efforts to better understand and address the challenges associated with food allergies. To prevent allergic reactions, individuals with food allergies must refrain from consuming food products that contain the allergen responsible for their allergic response [24]. The exact cause for this increase is not fully understood; however, it is attributed to shifts in lifestyle, environmental factors, and the process of modernization [25]. Furthermore, epidemiological data reveals a sexual dimorphism in food allergies, indicating that women are more susceptible to the condition [26]. However, our findings showed that male are more susceptible to the food allergies. In a 4 years multi-centre study conducted by Luo et al., the analysis revealed a statistically significant higher prevalence of sensitisation to nine allergens in males compared to that in females [27], while our results demonstrated that 23 types of allergens showed a statistically higher prevalence among males.

A recent study suggested that lower maternal vitamin D levels and insufficient vitamin D in infants are associated with an increased incidence of food allergies [28]. Gupta et al reported that a minimum of 10.8% (over 26 million) of adults in the United States have food allergies, while nearly 19% of adults believe they have a food allergy [29]. These findings emphasize the importance of providing adults with suspected food allergies with appropriate confirmatory testing and counseling. Burney et al demonstrated that the prevalence of IgE sensitization to foods ranged from 23.6% to 6.6% in European adults [30]. The authors showed that hazelnut, peach, and apple were the most commonly associated allergens. Besides, a recent systematic review implicated that the lifetime prevalence of food allergy in Europe has slightly increased since the previous review was published in 2014 [31]. Peanut allergies stand out as one of the most prevalent, severe, and persistent food allergies among all food allergies [32].

Alotiby et al. conducted a descriptive, cross-sectional study based on surveys, revealing that eggs, seafood, and fruits are the three primary food allergens in Saudi Arabia [33]. The authors suggested that their findings of may be help the government in making informed decisions about the components of meal planning in the future. In a survey conducted by Wu et al., based on questionnaires, it was found that less than 10% of the population in Taiwan experiences food allergies, with varying allergic symptoms and food allergens observed across different age groups [34]. The authors reported that mango, milk, peanuts, and eggs were identified as significant food allergens within the general population. Among children, milk, shellfish, peanuts, and eggs were commonly reported as allergens. Chan et al. reported that seafood, fish, and fruits are frequently implicated in causing acute allergic reactions in Taiwan [35]. While the majority of food allergies are mild, approximately 1% of patients may experience anaphylactic shock. Indeed, our findings showed that seafood (crab, clam, and shrimp) were one of the most common allergens in central Taiwan. Therefore, the emergency physicians should aware of the potential for food allergies, in particular those to seafood, to lead to anaphylactic shock, in central Taiwan. In addition, physicians should aware of the rising prevalence of other food allergies, notably those to peanut allergies, in Taiwan. In an epidemiological study conducted by Su et al., it was found that there is an increasing trend in the prevalence of food allergies, with rates of 10.4% in children and 12.5% in adults. Peanut allergies specifically showed an increase to 1.1% [36]. Our findings also showed that peanuts were one of the ten most common allergens identified, inducing sensitization in our study population.

Animal allergen exposure constitutes a significant risk factor for sensitization and the development of allergic diseases such as asthma, allergic rhinitis/conjunctivitis, and atopic dermatitis [5, 37]. Allergens can be effectively disseminated into the environment through the shedding of animal hair and dander, as well as the secretion and excretion of fluids. In certain professions, workers are frequently exposed to animal dander for extended periods, which can result in allergies. Several studies reported that work-related allergic symptoms in individuals working with animals, such as animal laboratory workers (30% with cat allergies, 25% with dog allergies) and veterinarians (26% with cat allergies, 19% with dog allergies), particularly during direct animal handling [37–39]. The primary sources of dog allergens are hair/dander and saliva. Also, cat dander contains several allergens. Dong et al implicated that exposure to pets in children without a predisposition to allergies was linked to an elevated susceptibility to respiratory diseases, with girls showing a higher susceptibility compared to boys [40]. According to our results, we demonstrated that females had a statistically higher rate of dog dander and cat dander allergen-specific sensitisation compared to males. In a 4-year multi-centre study conducted by Luo et al., the analysis revealed a statistically significant higher prevalence of sensitisation to nine allergens in males compared to that in females [27], while our results demonstrated that 23 types of allergens showed a statistically higher prevalence among males.

The results of our study provide valuable insights into the allergen distribution patterns in central Taiwan. The prevalence of allergens such as *D. farinae* and *D. pteronyssinus* suggests a high burden of dust mite allergies, which are commonly associated with respiratory symptoms. Besides, the substantial presence of house dust allergens indicates the need for effective strategies to reduce indoor allergen exposure such as improving indoor environments. Also, the identification of cockroach mix allergens highlights the importance of addressing environmental factors that contribute to indoor allergen sensitization. Additionally, the detection of crab, clamp, and shrimp allergens signifies the significance of specific food allergies in central Taiwan. We strongly believe that our findings contribute to an improved understanding of the epidemiology of allergies in our region and can guide public health interventions and clinical management approaches. Moreover, the main limitation of this study was that it was conducted in a single local district hospital; thus, the results may not be generalisable to patient populations in other hospitals and countries. While the data may not have global implications, it is still relevant and important for healthcare providers to understand the trends and patterns of allergen sensitisation in their own communities. Future research should prioritize the integration of clinical data, including comprehensive patient symptom profiles, treatment responses, and long-term follow-up data, to demonstrate a relationship between allergen exposures and patient outcomes.

In summary, this retrospective analysis provides valuable insights into the distribution patterns of allergens in central Taiwan over a 5 years period. The identification of prevalent allergens, including dust mites, house dust and specific food allergens, enhances our understanding of the local epidemiology of allergies. These findings can inform preventive measures, diagnostic strategies and therapeutic interventions to address the burden of allergic diseases in our region. However, further research is warranted to explore the underlying factors contributing to allergen sensitization and to develop targeted interventions for allergy prevention and management in central Taiwan.

## Summary Table

### What Is Known About This Topic

• Allergy is a type I hypersensitivity reaction to various allergens, including environmental factors, food, and drugs.
• Understanding the distribution patterns of allergens in specific regions is crucial for managing and preventing allergies effectively.
• Allergen-specific IgE testing is a common method to identify sensitization to specific allergens.

### What This Work Adds

• The study involved 8,444 patients in Central Taiwan who underwent allergen sensitization testing using the OPTIGEN^®^ Allergen-Specific IgE Assay from 2018 to 2022.
• Among food allergens, crab, clam, shrimp, peanut, and beef were the five most common.
• Gender-based variations in allergen prevalence were observed, with certain allergens showing higher prevalence among males and others among females.

This work represents an advance in biomedical science because it enhances our understanding of allergen distribution patterns in Central Taiwan, providing valuable insights into the epidemiology of allergies in the region and aiding in the development of targeted interventions and healthcare strategies.

## Data Availability

The original contributions presented in the study are included in the article/supplementary material, further inquiries can be directed to the corresponding author.
